# Current and Emerging Sedation Practices for Colonoscopy: A Narrative Review of Pharmacological Agents, High-Risk Populations, and Safety Considerations

**DOI:** 10.3390/jcm14186583

**Published:** 2025-09-18

**Authors:** Kamil Chudziński, Konstanty Szułdrzyński, Miłosz Jankowski, Kamil Adamczyk

**Affiliations:** Department of Anesthesiology and Intensive Care, National Medical Institute of the Ministry of the Interior and Administration, 02-507 Warsaw, Poland; kamil.chudzinski@pimmswia.gov.pl (K.C.); konstanty.szuldrzynski@pimmswia.gov.pl (K.S.); milosz.jankowski@pimmswia.gov.pl (M.J.)

**Keywords:** colonoscopy, anesthesia, sedation, propofol, endoscopy

## Abstract

Colonoscopy is the gold standard for colorectal cancer screening and diagnosis of gastrointestinal disorders, yet the procedure can still provoke anxiety and pain in many patients. Advances in anesthesia and sedation techniques have significantly improved patient tolerance while maintaining procedural efficiency and safety. This review explores the physiological mechanisms of pain during colonoscopy, compares anesthetic and sedative agents—including newer drugs like remimazolam and dexmedetomidine—and evaluates emerging evidence from recent studies on sedation efficacy, safety, and patient outcomes. Special attention is given to high-risk patient populations, including those with obesity, obstructive sleep apnea, cardiovascular diseases, respiratory disorders and frailty syndrome. Propofol-based sedation remains the most commonly used agent for deep sedation. However, newer pharmacological agents with enhanced pharmacokinetic properties and improved safety profiles are increasingly influencing contemporary anesthesia practices. An individualized approach to sedation is essential. Incorporating current evidence into clinical decision-making optimizes both patient experience and procedural outcomes.

## 1. Introduction

Colonoscopy is a cornerstone of modern gastroenterology and is essential for early colorectal cancer detection, polyp removal, and inflammatory bowel disease management. Despite its crucial role, patient apprehension remains high due to discomfort, bloating, and procedural anxiety. The challenge for anesthesiologists and gastroenterologists is to balance effective sedation with safety, particularly in vulnerable populations [1].

Pain during colonoscopy arises from multiple physiological factors, including colonic distension, looping of the colonoscope, mucosal irritation, and therapeutic interventions such as polypectomy. Selecting the appropriate sedative or anesthetic agent requires consideration of patient comorbidities, pharmacokinetics, recovery time, and procedural complexity. While traditional benzodiazepine–opioid regimens remain widely used, the shift toward propofol-based sedation and novel agents like remimazolam has been supported by growing evidence from large-scale studies [1,2].

This review provides an in-depth analysis of the mechanisms of pain in colonoscopy, compares different anesthetic regimens, highlights recent meta-analyses on sedation efficacy and safety, and discusses sedation strategies for special patient populations.

## 2. Materials and Methods

This narrative review synthesizes evidence on pharmacological strategies for colonoscopy sedation. A targeted, non-systematic search of PubMed/MEDLINE, Embase, and Web of Science was undertaken in March 2025 using the core string colonoscopy AND (sedation OR anesthesia) in combination with the agent names “midazolam”, “propofol”, “dexmedetomidine”, “remimazolam”, “ketamine”, and “lidocaine”. The search encompassed a window from January 1980 to March 2025. The search was limited to articles published in English.

Two independent reviewers (K.C. and K.A.) screened titles and abstracts to identify adult human studies reporting on clinical outcomes, pharmacodynamic/pharmacokinetic parameters, or safety endpoints. We excluded pediatric studies, animal experiments, opinion pieces, and conference abstracts. To ensure comprehensive coverage, reference lists of pertinent articles and society guidelines were also hand-searched. The electronic search yielded 286 records, and after removing duplicates and irrelevant papers, 64 full-text articles were deemed sufficiently informative to form the evidentiary basis of this review. Given the narrative design, a formal risk of bias assessment was not conducted; however, precedence was given to randomized controlled trials and large prospective cohorts in the interpretation of findings.

## 3. Mechanisms of Pain During Colonoscopy

**Figure 1:**
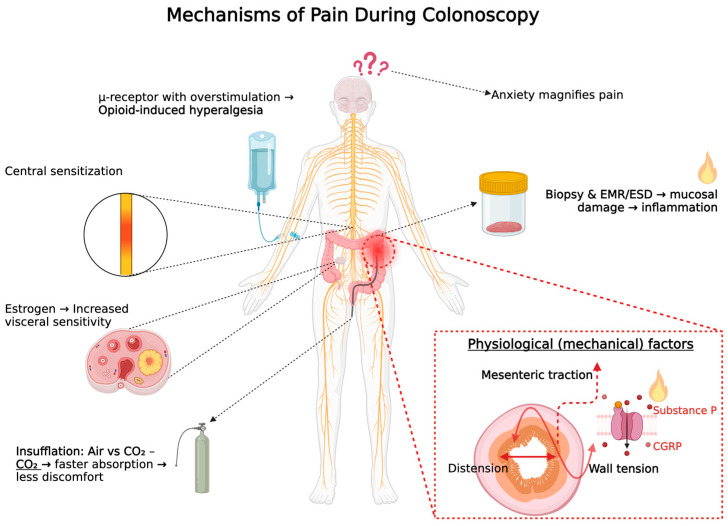
Multidimensional Mechanisms of Pain During Colonoscopy.

Despite the clinical value of colonoscopy, the experience can be uncomfortable and painful for some patients. Pain perception during colonoscopy is influenced by physiological, anatomical, and psychological factors. Understanding the underlying mechanisms of pain can lead to improved procedural techniques and patient outcomes [3]. Figure 1 illustrates the key mechanisms contributing to pain during colonoscopy, including both peripheral and central pathways.

The figure illustrates the principal mechanisms responsible for pain during colonoscopy. Peripheral contributors include colonic distension from insufflation (more pronounced with air than with carbon dioxide, CO_2_), mesenteric traction due to loop formation, and direct mucosal injury during biopsy or resection techniques such as EMR (endoscopic mucosal resection) and ESD (endoscopic submucosal dissection). Neurogenic inflammation is represented by the release of pain-amplifying neuropeptides—substance P and CGRP (calcitonin gene-related peptide). Central mechanisms are also shown, including spinal hyperexcitability (central sensitization), hormonal modulation (e.g., estrogen), and opioid-induced hyperalgesia (OIH). The figure also highlights the role of psychological factors, such as anxiety and anticipation, which modulate pain perception via cortical and limbic pathways.

### 3.1. Physiological Basis of Pain Perception

Pain experienced during colonoscopy primarily originates from the visceral nervous system. The colon lacks dense somatic innervation but is rich in visceral nociceptors, which respond to mechanical stimuli such as stretching, distension, and traction. The primary pain mechanisms include:

1. Colonic Distension: The insufflation of air or CO_2_ to visualize the colonic mucosa leads to distension, which activates mechanosensitive afferent fibers. These afferents transmit signals via the splanchnic and pelvic nerves, leading to discomfort and pain [3,4],
2. Mesenteric Traction: Excessive looping of the colonoscope can stretch the mesentery, activating mechanoreceptors in the visceral peritoneum. This form of pain is often described as deep and poorly localized [5],
3. Intestinal Wall Tension: The colon’s ability to stretch varies among individuals, influenced by age, sex, previous surgeries, and underlying conditions like irritable bowel syndrome (IBS) or inflammatory bowel disease (IBD). Increased wall tension can heighten visceral sensitivity [4,5,6],
4. Neurogenic Inflammation: In some patients, repeated stimulation of nociceptors may lead to the release of inflammatory mediators such as substance P and calcitonin gene-related peptide (CGRP). These neuropeptides enhance pain sensitivity by promoting local inflammation and neuronal excitability [6,7].

### 3.2. Neural Pathways and Pain Modulation

Pain perception during colonoscopy is mediated by a complex interaction between peripheral and central pain processing pathways. These include:

1. Visceral Afferent Pathways: The primary sensory inputs from the colon travel via the pelvic and splanchnic nerves to spinal segments T_10_-L_2_ and S_2_-S_4_. From there, they ascend to the brainstem and thalamus, where they are relayed to higher cortical centers involved in pain perception [8],
2. Spinal and Supraspinal Processing: Within the spinal cord, nociceptive signals undergo modulation by excitatory and inhibitory neurotransmitters. In some individuals, hyperexcitability of dorsal horn neurons may contribute to heightened pain responses, a phenomenon known as central sensitization [9],
3. Cortical and Subcortical Processing: The anterior cingulate cortex, insula, and prefrontal cortex play key roles in integrating pain-related information with emotional and cognitive factors. Anxiety and anticipation of pain can lead to increased activity in these regions, enhancing pain perception [4,5,10].

### 3.3. Factors Influencing Pain Severity

Several factors contribute to variations in pain experience during colonoscopy:

1. Patient-Related Factors:
  - Anxiety and Psychological State: Higher levels of pre-procedural anxiety correlate with increased pain perception due to the influence of the limbic system on pain processing [9,10],
  - Sex Differences: Women generally report higher pain scores than men, potentially due to hormonal influences on visceral sensitivity [10],
  - Age and Comorbidities: Older adults may have reduced visceral sensitivity, whereas patients with IBS, IBD, or prior abdominal surgeries may experience heightened pain due to altered pain processing. Patients with IBD, such as Crohn’s disease and ulcerative colitis, have inflamed mucosa with heightened sensitivity, making them more prone to pain during endoscopic contact. The presence of ulcers, erosions, or prior radiation damage can further increase pain perception due to exposure of nerve endings and altered mucosal integrity. Patients with chronic opioid use may experience increased pain during the colonoscopy due to opioid-induced hyperalgesia [3,10,11].
2. Colonic Anatomical Variability:
  - Colon Length and Mobility: Redundant or elongated colons, commonly seen in women and elderly patients, can increase the risk of looping and mesenteric traction [12],
  - Previous Surgeries and Adhesions: Postoperative adhesions can alter colonic mobility, leading to increased resistance during scope insertion and higher pain levels [10,11,12,13].
3. Endoscopic Technique:
  - Scope Insertion and Loop Formation: Excessive looping of the colonoscope within the sigmoid or transverse colon can generate unnecessary stretching forces, exacerbating pain [12],
  - Insufflation Type: While air insufflation is traditionally used, CO_2_ insufflation has been found to cause less discomfort due to its rapid absorption from the bowel [13],
  - Withdrawal Techniques: Gentle maneuvering of the scope during withdrawal minimizes unnecessary tension on the colonic wall, reducing pain [12,13],
  - Polypectomy and biopsy: It causes localized tissue trauma, leading to transient pain and an inflammatory response. Post-polypectomy coagulation syndrome occurs due to thermal injury extending into the colonic wall, resulting in transmural burns that can cause delayed visceral pain and mimic peritonitis [11],
  - Endoscopic mucosal resection (EMR) and endoscopic submucosal dissection (ESD)**,** which involve deeper tissue resection, can activate pain pathways more intensely than standard biopsy procedures [11].

Pain and discomfort experienced during colonoscopy may necessitate premature termination of the procedure before complete visualization of the colon, increasing the risk of missing neoplastic lesions. Additionally, negative experiences may discourage patients from undergoing future screenings, potentially compromising early detection and prevention efforts. Therefore, implementing strategies to mitigate procedural discomfort is crucial for enhancing patient compliance and ensuring high-quality colonoscopic examinations. One of the most effective approaches to achieving this goal is the use of appropriate sedation or anesthesia, which has been shown to enhance patient tolerance and procedural success rates [1,2,11,12].

## 4. Sedation and Anesthesia for Colonoscopy

### 4.1. General Considerations

The choice of sedation or anesthesia for colonoscopy varies worldwide, depending on healthcare system resources, medical staff availability, procedural indications, and local practices. In many countries colonoscopies are frequently performed without sedation. Legal regulations influence who can administer sedation, ranging from anesthesiologists to specially trained nurses or endoscopists on their own. While colonoscopy is predominantly performed on an outpatient basis, hospitalization may be required for high-risk patients due to anesthesia-related or procedural risks [12,14].

Appropriate sedation in colonoscopy alleviates anxiety, minimizes pain, and enhances both patient and operator comfort. By reducing discomfort, sedation can improve procedural efficiency, facilitate complete visualization of the colon and terminal ileum, and potentially increase the detection rate of dysplastic or neoplastic lesions. Furthermore, optimizing patient experience may improve adherence to future surveillance colonoscopies, which is critical for early colorectal cancer detection and prevention [2,12,14].

In 2020, expert consensus guidelines were published on sedation and anesthesia protocols for hospital-based colonoscopy. These recommendations emphasize the safety and benefits of sedation in enhancing patient satisfaction and procedural tolerance. For most patients, minimal to moderate sedation—where verbal communication remains possible throughout the procedure—is sufficient. Additional non-pharmacological strategies, such as pre-procedural education, verbal reassurance during the examination, CO_2_ insufflation instead of air, thermal comfort, music therapy, and the presence of a companion, may further enhance patient comfort [14].

The guidelines do not recommend routine deep sedation or general anesthesia for hospital-based colonoscopies. During deep sedation, patients experience reduced consciousness and diminished protective reflexes, although spontaneous respiration is preserved [2,14]. The use of general anesthesia should be reserved for selected patients, with careful consideration given to individualized risk-benefit assessment. Continuous monitoring of vital signs and anesthesiology oversight are essential in such cases. Standardized institutional protocols for sedation administration and patient monitoring are strongly encouraged [14].

Table 1 outlines key factors that may warrant consideration of deep sedation during colonoscopy. The presence of at least two of these factors should prompt a discussion regarding sedation strategy. Ultimately, the decision should be made collaboratively between the patient, endoscopist, and anesthesiologist [12,14].

The choice of anesthesia for colonoscopy should be tailored to individual patient needs, balancing sedation depth, safety, and recovery time. A variety of drugs are used for sedation and anesthesia during colonoscopy, with propofol, benzodiazepines (such as midazolam), and opioids (such as fentanyl) being the most frequently employed agents. In recent years, newer drugs like dexmedetomidine and remimazolam have gained attention due to their favorable pharmacokinetics and improved safety profiles. The following section provides a comparative analysis of these agents, highlighting their mechanisms, advantages, and limitations.

### 4.2. Pharmacological Agents Used in Colonoscopy Sedation and Anesthesia

**Figure 2:**
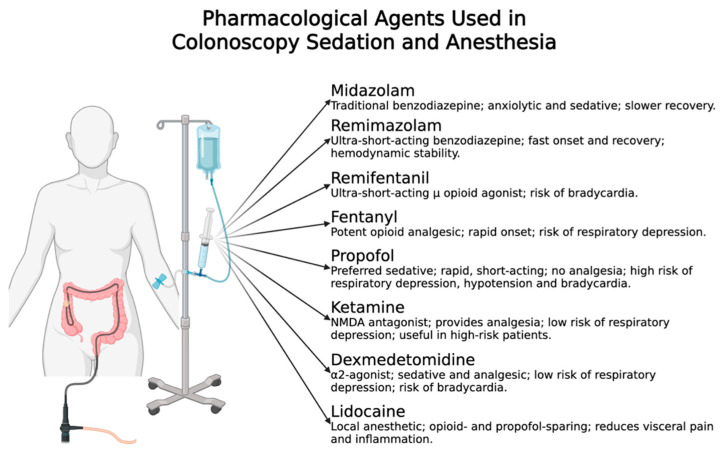
Pharmacological Agents Commonly Used in Colonoscopy Sedation and Anesthesia.

The pharmacological agents most commonly used for sedation and anesthesia during colonoscopy, along with their key properties and safety profiles, are summarized in Figure 2.

#### 4.2.1. Benzodiazepines

Benzodiazepines are widely utilized for sedation during colonoscopy due to their anxiolytic, amnestic, and muscle-relaxant properties. Their primary mechanism of action involves potentiation of γ-aminobutyric acid (GABA) at GABA-A receptors, leading to dose-dependent sedation and anxiolysis without analgesic effects [14,15,16].

##### Midazolam

Midazolam remains the most commonly used benzodiazepine in endoscopic procedures due to its rapid onset (2–5 min) and short duration of action (30–60 min). It undergoes hepatic metabolism via cytochrome P450 enzymes and is eliminated renally. The typical dosing regimen consists of 0.02–0.1 mg/kg IV as a bolus, with additional increments as needed to achieve the desired level of sedation. Despite its advantages, midazolam has limitations, including variable duration of sedation, cumulative effects with repeated dosing, and delayed recovery in elderly or hepatic-impaired patients [15,16].

##### Remimazolam

Remimazolam is a new-generation, ultra-short-acting benzodiazepine that is rapidly metabolized by non-specific tissue esterases, offering a more predictable pharmacokinetic profile. Compared to midazolam, it provides a faster onset (1–2 min) and shorter recovery time, with reduced risk of prolonged sedation. The recommended dosing involves an initial IV bolus of 2.5–5 mg, followed by additional titrated doses (1.0–2.5 mg) as needed. Remimazolam’s advantages include rapid clearance, minimal accumulation, and availability of flumazenil as a reversal agent, making it a promising alternative in patients requiring short procedural sedation with rapid recovery. However, its higher cost and limited real-world data remain considerations [16].

While both midazolam and remimazolam are effective for moderate sedation, remimazolam may offer advantages in procedures requiring rapid patient recovery and hemodynamic stability. Further comparative studies are necessary to establish its long-term clinical benefits over traditional benzodiazepines [16].

#### 4.2.2. Opioids

Opioids are frequently utilized in gastrointestinal endoscopy, particularly in combination with benzodiazepines or propofol, to enhance patient comfort by providing potent analgesia and mild sedation. Among them, fentanyl is the most commonly used agent due to its rapid onset, short duration of action, and high analgesic potency. However, concerns regarding opioid-related adverse effects, the ongoing opioid crisis, and the impact of chronic opioid use in patients with conditions such as IBD have led to increased scrutiny regarding their role in procedural sedation [15,17,18].

##### Fentanyl

Fentanyl is a synthetic μ-opioid receptor agonist with approximately 100 times the potency of morphine. It exhibits a rapid onset (1–3 min) and short duration (30–60 min), making it particularly suitable for procedural sedation, where quick recovery is desired. The typical intravenous dose ranges from 25 to 100 µg, with titration based on patient response. Compared to longer-acting opioids, fentanyl is associated with reduced post-procedural drowsiness but carries a risk of dose-dependent respiratory depression, chest wall rigidity, and nausea [15,17].

In patients undergoing colonoscopy, fentanyl is frequently combined with benzodiazepines (e.g., midazolam) or propofol to achieve adequate sedation and pain control while allowing for rapid post-procedural recovery. However, due to interindividual variability in opioid sensitivity, particularly in elderly patients or those with hepatic or renal impairment, careful dose titration and patient monitoring are necessary [15].

While fentanyl provides effective pain relief, its routine use in endoscopic sedation has come under increasing scrutiny due to concerns about opioid dependence, chronic opioid use, and the ongoing opioid crisis [18].

The global opioid crisis has highlighted the risks of opioid overprescription, dependence, and associated morbidity, leading to increased efforts to adopt opioid-sparing strategies in procedural sedation. Repeated opioid exposure, even in the context of procedural use, has the potential to contribute to tolerance, hyperalgesia, and dependence, particularly in vulnerable populations [18].

One group at particular risk includes patients with chronic opioid use, such as those with IBD (Crohn’s disease and ulcerative colitis). Those patients frequently suffer from chronic abdominal pain, leading to high opioid prescription rates. However, long-term opioid therapy in this population has been linked to worse disease outcomes, increased risk of hospitalization, opioid-induced bowel dysfunction, and opioid-induced hyperalgesia [18,19].

For these patients, opioid-based sedation may result in prolonged recovery times, increased respiratory depression, and altered pain sensitivity, necessitating cautious dosing and consideration of alternative sedation protocols. Strategies such as multimodal analgesia (e.g., dexmedetomidine, lidocaine, or ketamine) and patient-centered opioid de-escalation approaches should be prioritized to improve procedural safety and long-term patient outcomes [15,18].

##### Remifentanil

Remifentanil, an ultra-short-acting μ-opioid agonist, is typically administered as a continuous intravenous infusion and can also be delivered via target-controlled infusion (TCI) for precise titration. For colonoscopy sedation, low-dose regimens commonly start with a 0.5 µg/kg IV bolus followed by a continuous infusion of 0.025–0.125 µg/kg/min, titrated to effect. In a prospective, randomized study comparing low-dose remifentanil infusion with propofol, remifentanil provided superior analgesia and patient comfort, required fewer supplemental doses, and facilitated rapid recovery, while maintaining stable hemodynamics and end-tidal CO_2_ levels. However, sedation depth was lighter than with propofol; moreover, remifentanil may induce chest wall rigidity, which can complicate ventilation, particularly with rapid or high-dose administration [15,20,21].

#### 4.2.3. Propofol

Propofol, a short-acting intravenous anesthetic, is a highly lipophilic GABA-A receptor agonist that also inhibits N-methyl-D-aspartate (NMDA) receptors and modulates calcium ion flux through slow channels, primarily within the reticular formation [22].

It has become the preferred sedative agent for gastrointestinal endoscopy, including colonoscopy, due to its rapid onset (30–60 s), short duration of action (5–10 min), and favorable recovery profile. The depth of sedation induced by propofol is dose-dependent. At low doses (0.5–1 mg/kg), it exerts a sedative effect. Additionally, like other GABA-A receptor agonists, it possesses anticonvulsant and antiemetic properties. However, propofol lacks analgesic effects. Compared to traditional opioid–benzodiazepine regimens, propofol-based sedation has been associated with superior patient tolerance, reduced procedural discomfort, and decreased recovery time, thereby enhancing both procedural efficiency and patient satisfaction. However, its use necessitates careful dose titration and continuous monitoring due to the risk of dose-dependent cardiorespiratory depression [14,15,22].

Propofol is typically administered via intravenous (IV) bolus injection (0.5–1 mg/kg IV) or, less preferably, continuous infusion, with dosing individualized according to patient age, comorbidities, and procedural requirements [14,15,22].

Alternatively, target-controlled infusion (TCI) of propofol provides more stable sedation and fewer agitation episodes than repeated boluses, with a similar safety profile, though it may increase total propofol dose and slightly prolong recovery [23]. When combined with remifentanil via TCI, this approach further reduces propofol requirements and lowers the incidence of respiratory depression compared with manually controlled techniques [21].

Table 2 presents the dosing of propofol for sedation during colonoscopy in specific patient groups [22,23].

In patients with advanced age, cardiopulmonary disease, or obesity, lower induction doses and slower infusion rates are recommended to minimize the risk of hypotension and respiratory depression [15,22].

While propofol is generally well tolerated, its use is associated with several dose-dependent adverse effects, including:

- Respiratory depression and apnea (most common dose-limiting effect)
- Hypotension and bradycardia (secondary to systemic vasodilation)
- Injection site pain (mitigated by co-administration of lidocaine) [22].

#### 4.2.4. Ketamine

Ketamine, a phencyclidine derivative, is a unique dissociative anesthetic with potent analgesic, amnestic, and sedative properties. Its primary mechanism of action is NMDA receptor antagonism, which disrupts pain transmission and induces a dissociative state. Additionally, ketamine exerts opioid receptor agonism and inhibits catecholamine reuptake within the central nervous system, further enhancing its analgesic effects. Unlike conventional GABA-A receptor agonists ketamine preserves spontaneous respiration and airway reflexes, making it particularly advantageous for procedural sedation [24].

The use of ketamine for colonoscopy sedation has gained interest due to its analgesic properties, cardiovascular stability, and respiratory-sparing effects. In contrast to traditional sedatives, ketamine provides adequate analgesia without inducing significant respiratory depression, making it an alternative for high-risk patients, such as those with cardiopulmonary comorbidities or opioid tolerance [24].

Studies suggest that low-dose ketamine (0.1–0.5 mg/kg IV), administered either alone or in combination with agents such as propofol or benzodiazepines, enhances patient comfort while maintaining hemodynamic stability. Co-administration of ketamine with propofol allows for dose reduction in both agents, minimizing the risk of hypotension and respiratory depression commonly associated with propofol monotherapy [15,24].

Despite its advantages, ketamine is associated with dose-dependent psychomimetic effects, including hallucinations, dysphoria, and dissociation, which may limit its routine use in ambulatory settings. The incidence of these adverse effects is dose-related and more pronounced at higher anesthetic doses. However, co-administration of benzodiazepines or propofol has been shown to attenuate these reactions, improving patient tolerance [24].

Other potential adverse effects include sympathomimetic responses, such as increased blood pressure and heart rate, which may be beneficial in hemodynamically unstable patients but require caution in individuals with uncontrolled hypertension or coronary artery disease [24].

#### 4.2.5. Dexmedetomidine

Dexmedetomidine is a highly selective α_2_-adrenergic receptor agonist that provides sedation, anxiolysis, and analgesia without significant respiratory depression. Unlike traditional sedatives such as propofol and benzodiazepines, it induces a state of natural sleep-like sedation, characterized by easy arousability and preservation of spontaneous breathing. Its mechanism involves the inhibition of norepinephrine release in the locus coeruleus, leading to a dose-dependent reduction in sympathetic tone, decreased heart rate, and mild analgesic effects [25].

Dexmedetomidine has gained attention as an alternative or adjunct to conventional sedation techniques for colonoscopy, particularly in patients at higher risk of respiratory depression, such as those with chronic obstructive pulmonary disease (COPD) or obstructive sleep apnea (OSA) [15,25,26].

Studies suggest that intravenous loading doses of 0.5–1 μg/kg over 10 min, followed by a continuous infusion of 0.2–0.7 μg/kg/h, provide effective sedation while maintaining cardiorespiratory stability. Dexmedetomidine is often used in combination with propofol, ketamine or opioids, allowing for dose reduction in these agents and minimizing their respective adverse effects [25,26].

A key advantage of dexmedetomidine over other sedatives is its minimal effect on respiratory function, making it particularly useful for patients with preexisting pulmonary disease or those requiring prolonged procedures. Additionally, its opioid-sparing properties make it beneficial for individuals with opioid tolerance or chronic pain syndromes [18,25].

However, dexmedetomidine has notable hemodynamic effects, including bradycardia and hypotension, due to its sympatholytic properties. These effects are usually dose-dependent and more pronounced in elderly patients or those with preexisting cardiac disease. Unlike propofol, dexmedetomidine has a longer onset time and a prolonged recovery period, which may limit its use in fast-paced ambulatory endoscopy settings [25,26].

#### 4.2.6. Lidocaine

Lidocaine, an amide-type local anesthetic, exerts its primary pharmacological effects through the inhibition of voltage-gated sodium channels, thereby preventing neuronal depolarization and impulse propagation. This mechanism underlies its dual role as both a local anesthetic and an antiarrhythmic agent. Additionally, lidocaine modulates the glycinergic system and, at higher concentrations, acts as an inhibitor of potassium and calcium channels, including NMDA receptors, contributing to its broad pharmacodynamic profile. When administered intravenously, lidocaine enhances the effects of anesthetic agents, reduces central sensitization-induced hyperalgesia, and mitigates opioid-induced hyperalgesia. Its multimodal effects include analgesic and anti-inflammatory properties. Lidocaine reduces inflammatory cytokine release and mitigates peripheral and central sensitization, which may be particularly beneficial for patients undergoing painful endoscopic procedures. It has a rapid onset of action (typically within 45–90 s after intravenous administration), with a half-life of approximately 1.5 to 2 h. It undergoes hepatic metabolism with renal excretion of its metabolites [27].

Intravenous lidocaine enhances respiratory center sensitivity to CO_2_ levels, while also reducing laryngeal and bronchial reactivity, which may lower the risk of airway complications during sedation [28]. Lidocaine has been shown to promote intestinal peristalsis, reducing the risk of postoperative ileus. It may exert mild cardiodepressant effects. Excessive dosing can result in systemic toxicity, typically presenting as hypotension and bradycardia, and, in severe cases, progressing to cardiac arrest. Neurological symptoms, including convulsions, have been reported at an average dose of approximately 8 mg/kg, corresponding to a plasma concentration of around 15 μg/mL. The earliest signs of cardiotoxicity generally occur at plasma concentrations exceeding 21 μg/mL [27,29].

The use of intravenous lidocaine in colonoscopy has gained interest due to its opioid-sparing effects, particularly in the context of the ongoing opioid crisis. Lidocaine infusions (typically a bolus of 1–1.5 mg/kg followed by a continuous infusion of 1–2 mg/kg/h) have been reported to reduce pain perception and procedural discomfort, potentially enhancing patient tolerance. Moreover, its visceral analgesic effects are especially advantageous for individuals with IBD, who often experience heightened visceral sensitivity and exaggerated nociceptive responses [27,29,30].

Lidocaine has a wide therapeutic index and is generally well tolerated. However, systemic toxicity may occur with excessive plasma concentrations, particularly in elderly or malnourished patients or those with comorbidities. Early symptoms of lidocaine toxicity include metallic taste, tinnitus, perioral numbness, visual disturbances, dizziness and headache. In severe cases, neurotoxicity can progress to seizures, altered consciousness, or coma, while cardiovascular toxicity may result in profound hypotension, bradycardia, and cardiac arrest. The risk of adverse events underscores the importance of appropriate dosing, close monitoring, and cautious patient selection when incorporating lidocaine into procedural sedation protocols [27,29].

### 4.3. Comparative Efficacy and Safety of Sedation Regimens

The selection of an optimal sedation regimen for colonoscopy requires a nuanced understanding of the comparative benefits and risks of available pharmacological agents. While propofol-based sedation is established as a highly effective standard, its narrow therapeutic index necessitates a critical evaluation of alternative and combination regimens, particularly in high-risk populations. This section provides a critical synthesis of the evidence, comparing key agents and regimens based on clinical endpoints to guide individualized decision-making.

#### 4.3.1. Hemodynamic Stability

The traditional “balanced sedation” with midazolam and fentanyl carries a significant risk of synergistic hypotension, a key concern in vulnerable patients [31]. This regimen, while effective, often results in less predictable hemodynamic responses compared to newer agents. Propofol’s principal limitation remains its dose-dependent hypotension, a risk well-documented in large-scale analyses of colonoscopy procedures [31,32].

In contrast, remimazolam consistently demonstrates superior hemodynamic stability. A comprehensive meta-analysis by Koo et al. (2025) comparing remimazolam to propofol for colonoscopy found a significantly lower risk of hypotension with remimazolam [33]. Another meta-analysis focusing specifically on elderly patients confirmed these findings, highlighting remimazolam’s particular suitability for this high-risk group, with significantly reduced risks of bradycardia and hypoxemia [34].

Propofol-sparing strategies offer a pragmatic compromise for maintaining hemodynamic stability. The addition of low-dose esketamine has been shown to significantly reduce the incidence of hypotension during propofol-based sedation for colonoscopy. A systematic review and meta-analysis by Kan et al. (2024) demonstrated that esketamine combinations significantly reduced the risk of hypotension and bradycardia [35].

#### 4.3.2. Respiratory Safety

The combination of a benzodiazepine and an opioid is notorious for causing respiratory depression due to synergistic effects [36]. Propofol also carries a high, dose-dependent risk of apnea. In contrast, both remimazolam and dexmedetomidine exhibit markedly better respiratory safety profiles [37,38].

The meta-analysis by Koo et al. (2025) confirmed a significantly lower incidence of respiratory depression with remimazolam compared to propofol in colonoscopy patients, with respiratory depression occurring in only 3.6% of remimazolam patients versus 13.0% in the propofol group [33]. Another systematic review comparing remimazolam to midazolam for procedural sedation also found remimazolam to be associated with fewer respiratory adverse events [39].

Dexmedetomidine provides sedation without clinically significant respiratory depression, making it a valuable agent for patients with underlying respiratory disease such as obstructive sleep apnea (OSA) or chronic obstructive pulmonary disease (COPD). A meta-analysis by Tang et al. (2023) confirmed the favorable respiratory safety profile of dexmedetomidine in gastrointestinal endoscopic procedures [40].

#### 4.3.3. Recovery Profile and Cognitive Outcomes

A major drawback of the midazolam/fentanyl combination is its prolonged and unpredictable recovery, along with a higher risk of postoperative cognitive dysfunction (POCD) [41]. While propofol offers rapid recovery, concerns about POCD persist, particularly in elderly patients [42].

Remimazolam demonstrates a significant advantage in recovery profiles, with studies consistently showing faster and more complete cognitive recovery compared to both midazolam and propofol. The Koo et al. meta-analysis found significantly shorter emergence times and post-procedural unit stay times with remimazolam compared to propofol [33]. Recent studies have also demonstrated remimazolam’s potential to reduce the incidence of POCD through mechanisms involving reduced neuroinflammation [43,44].

Dexmedetomidine has been associated with a lower incidence of postoperative delirium and may offer neuroprotective effects, particularly in elderly patients [45,46].

#### 4.3.4. Analgesia, Procedural Conditions, and Satisfaction

The satisfaction of both the patient and the endoscopist is a critical determinant of a successful regimen. Propofol monotherapy consistently achieves high patient satisfaction due to its rapid onset and smooth recovery [47]. However, its lack of analgesia can lead to patient movement, potentially compromising procedural conditions for the endoscopist.

The addition of a potent, short-acting opioid like fentanyl significantly improves procedural conditions by suppressing visceral pain responses. This results in an immobile patient, which is highly valued by endoscopists and often translates to the highest satisfaction scores for this group, despite the increased respiratory risk [48]. Song et al. (2024) compared patient-controlled propofol-remifentanil sedation with total intravenous propofol-midazolam-fentanyl anesthesia, demonstrating equivalent analgesic efficacy while the remifentanil regimen offered superior recovery profiles and enhanced hemodynamic stability [49].

Propofol-ketamine combinations demonstrate superior patient satisfaction scores compared to propofol-fentanyl regimens [50]. In contrast, dexmedetomidine, despite maintaining a good safety profile without increased risk of hypotension and hypoxia, causes significant heart rate reduction and diminished patient satisfaction, though it provides comparable suppression of body movements and gagging reflexes to propofol alone [51].

#### 4.3.5. Conclusion and Ideal Clinical Scenarios

No single sedation regimen is universally superior; the optimal choice is dictated by the patient’s clinical profile and procedural demands.

- For healthy, low-risk patients (ASA I-II), propofol monotherapy remains a highly effective option, providing rapid onset, excellent procedural conditions, and high patient satisfaction.
- For patients with significant cardiovascular risk, remimazolam emerges as the preferred agent due to its superior hemodynamic stability and significantly reduced risk of hypotension and bradycardia.
- For patients with severe respiratory disease (e.g., OSA, COPD), a regimen based on dexmedetomidine, remimazolam, or a propofol-ketamine combination offers the greatest margin of safety due to minimal respiratory depression.
- To achieve optimal procedural conditions for the endoscopist, especially during complex procedures, a combination of propofol with a short-acting opioid (fentanyl/remifentanil) or ketamine provides the most stable field and highest endoscopist satisfaction.
- The traditional midazolam-fentanyl regimen, given its less favorable safety and recovery profile, should be considered a second-line option when modern alternatives are unavailable or contraindicated.

## 5. Anesthesia-Related Complications in Colonoscopy: Risks, Management, and Advances in Sedation Strategies

While the procedure is generally safe, the administration of anesthesia introduces potential risks and complications. The safety profile of anesthesia varies based on the type of sedative or anesthetic used, patient comorbidities, and procedural factors. Understanding these risks is critical for optimizing patient outcomes, ensuring informed consent, and improving procedural safety [52,53,54].

Cardiopulmonary complications during colonoscopy are a known concern and can occur during the procedure or in the post-procedural period. The Clinical Outcomes Research Initiative (CORI) database has reported a complication rate from 0.9% to 6% of cases, depending on the sedative regimen and patient characteristics. Common cardiopulmonary events include low blood pressure (hypotension), slow heart rate (bradycardia), desaturation (hypoxemia), and respiratory depression [53].

Intraoperative and postoperative hypotension are frequent occurrences linked to organ injury (stroke, myocardial infarction and acute kidney injury during 30 days after procedure) and adverse outcomes. Fluctuations in arterial blood pressure during procedural sedation remain poorly characterized [55,56]. According to Sneyd et al.’s analysis, hypotension is a frequent complication during propofol sedation for colonoscopy, with 36% of patients experiencing episodes of low blood pressure. Their study found that higher doses of propofol and prolonged sedation were associated with more severe and longer-lasting hypotension [55].

A meta-analysis of 18 randomized controlled trials (Lieber) further confirmed the increased risk of hypotension with propofol compared to other sedatives. The risk was significantly higher than with etomidate, remimazolam, and midazolam. However, propofol was associated with a lower risk of hypotension compared to dexmedetomidine [56].

Studies have also reported higher incidences of heart attacks (myocardial infarction), angina, and strokes within 30 days post-colonoscopy, particularly in individuals with pre-existing heart disease, but further investigations are needed [53,54].

Risk assessment before colonoscopy is essential, particularly for patients with conditions such as hypovolemia (associated with bowel preparation before colonoscopy), hypertension, coronary artery disease, or heart failure. Utilizing tools such as the American Society of Anesthesiologists (ASA) classification can help identify high-risk individuals. Patients with significant cardiac risk factors may benefit from an anesthesiologist consultation and modified sedation protocols to reduce cardiovascular strain including a propofol-sparing strategy [16,30,52,53,55,57,58].

Given the high incidence of hypotension associated with propofol sedation for colonoscopy, propofol-sparing strategies have emerged as an approach to mitigate hemodynamic instability while maintaining effective sedation. The propofol-sparing effect refers to the reduction in total propofol dosage achieved through the concurrent use of adjunct medications, thereby minimizing its dose-dependent adverse effects, including hypotension and prolonged sedation.

Adjunct agents such as low doses of dexmedetomidine or ketamine, intravenous lidocaine, and benzodiazepines (particularly remimazolam) have been explored to reduce propofol requirements while preserving adequate sedation and patient comfort. Dexmedetomidine, an α2-adrenergic agonist, has demonstrated sedative and analgesic properties while maintaining respiratory stability; however, its association with bradycardia and hypotension necessitates careful patient selection [26,55]. Ketamine, an NMDA receptor antagonist, provides analgesia and sedation while stimulating the sympathetic nervous system, potentially counteracting the hypotensive effects of propofol [30,59]. Lidocaine, administered intravenously, has been shown to reduce propofol requirements by decreasing the pain response and enhancing sedation, while benzodiazepines such as midazolam can effectively reduce induction doses of propofol [57,58,60]. In clinical practice, the implementation of propofol-sparing strategies requires careful dose titration and monitoring to balance sedation depth, hemodynamic stability, and recovery time. Given the findings, incorporating these adjuncts may offer a means of reducing the incidence of procedural hypotension and improving overall patient safety during colonoscopy [55,57,58,59,60].

Adverse respiratory events are one of the most common complications during anesthesia for gastrointestinal endoscopy. These include apnea, decreased oxygen saturation of hemoglobin, and secondary hypoxemia. While most respiratory disturbances are transient and have no significant consequences, prolonged episodes or occurrences in medically compromised patients may lead to cardiac arrest and death [53,61,62].

Bhananker et al. analyzed malpractice claims related to sedation-related complications in the United States using ASA registries from 1990 to 2002. Excessive sedation leading to respiratory depression, hypoxia-induced severe central nervous system injury, and death were the most frequent complications forming the basis of these claims [63].

One strategy to reduce the risk of respiratory complications during anesthesia is to decrease the dose of propofol and/or avoid the use of opioids (opioid free anesthesia), as these agents are primary contributors to respiratory failure [14,15,17,19].

Opioid-free anesthesia (OFA) has emerged as a safer alternative in the management of procedural sedation, especially considering the global opioid crisis. With increasing concerns regarding opioid overuse and misuse, there is a growing push towards reducing opioid consumption in medical procedures, including colonoscopy. Opioids are often associated with respiratory depression, delayed recovery, and higher risks of addiction and overdose in vulnerable populations. By minimizing or eliminating opioid use, OFA provides a safer approach that can also help combat the broader public health challenge of opioid dependence [15,17,18,64].

The use of non-opioid agents such as dexmedetomidine, ketamine, lidocaine, and remimazolam has been shown to reduce the need for high-dose propofol while maintaining effective sedation, all while significantly lowering the risk of respiratory depression and hypoxia. These alternatives offer better airway safety and faster recovery times compared to traditional opioid-based sedation protocols. Furthermore, OFA aligns with current healthcare initiatives to reduce opioid exposure and dependency, providing both clinical and public health benefits in procedural sedation [17,18,64].

Research by Dalal et al. further supports the value of reducing opioid exposure, particularly in patients with IBD. Their study demonstrated that intravenous opioid exposure (even during sedation) in hospitalized patients with IBD was linked to future opioid use, leading to a higher risk of opioid dependence and long-term use in these patients [65]. By utilizing opioid-free anesthesia in colonoscopy procedures for IBD patients, we can mitigate the risk of future opioid dependence, improving clinical outcomes while aligning with broader strategies to combat the opioid crisis.

Thus, opioid-free anesthesia is not only an effective strategy to enhance patient safety but also a critical step toward mitigating the risks posed by the ongoing opioid epidemic, especially in populations at higher risk of opioid misuse.

The risk of severe respiratory complications associated with deep sedation for colonoscopy, specifically aspiration pneumonia, has remained unclear [53,54]. While some previous studies suggested an increased risk, more recent analyses have not demonstrated this association, nor have they shown an elevated risk of any infections within 30 days following colonoscopy under deep sedation/general anesthesia [54,66].

This warrants further investigation, particularly in the context of the increasing number of patients using glucagon-like-peptide type 1 (GLP-1) receptor agonists for weight loss or other indications. These medications delay gastric emptying, which may potentially increase the risk of aspiration during sedation or anesthesia [67].

Although rare, allergic reactions to sedation agents, including propofol, lidocaine, and opioids, can occur, ranging from mild hypersensitivity to life-threatening anaphylaxis. A history of drug allergies should be carefully reviewed before sedation [68,69].

Sedation for colonoscopy, particularly with benzodiazepines like midazolam, can lead to post-procedural cognitive impairment, especially in elderly patients and those with preexisting neurological conditions. Paradoxical reactions, including agitation, aggression, and disinhibition, may occur due to GABA receptor dysregulation, with risk factors such as advanced age, anxiety disorders, and chronic benzodiazepine use [70,71].

The studies show that adding low-dose ketamine or dexmedetomidine to propofol helps preserve cognitive function after sedation for colonoscopy, supporting their role in propofol-sparing and opioid-free strategies while maintaining effective sedation [72,73].

Remimazolam, due to its shorter half-life and rapid metabolism, may further reduce cognitive side effects compared to midazolam, but further studies are needed. A study by Lin et al. comparing remimazolam with propofol in elderly patients undergoing colonoscopy found that remimazolam was associated with faster cognitive recovery than propofol, suggesting it may offer a safer alternative for elderly patients while reducing the risk of cognitive impairment post-sedation [74].

Consequently, in high-risk patients, opioid-free anesthesia or propofol-based sedation with adjuvants may be preferred to optimize cognitive outcomes. Careful patient selection, dose adjustment, and multimodal sedation strategies are crucial for minimizing cognitive complications while ensuring procedural success.

## 6. High-Risk Patient Populations

**Figure 3:**
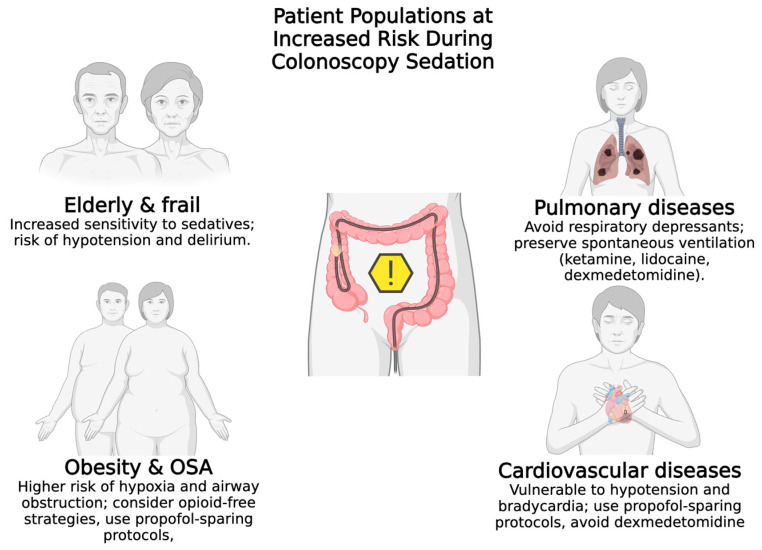
Patient Populations at Increased Risk During Colonoscopy Sedation.

Sedation during colonoscopy requires careful consideration of various patient-specific factors, particularly in populations with obesity, obstructive sleep apnea, cardiovascular or respiratory diseases, and elderly or frail individuals. The selection of an appropriate anesthetic agent and sedation strategy is crucial to minimize risks and optimize patient outcomes. Patient populations particularly vulnerable to sedation-related complications during colonoscopy are presented in Figure 3, which outlines the main risks and tailored anesthetic considerations for each group.

### 6.1. Obesity and Obstructive Sleep Apnea (OSA)

Morbidly obese patients and those with obstructive sleep apnea (OSA) present unique challenges during sedation due to airway collapsibility, reduced oxygen reserves, and increased sensitivity to respiratory depressants. Many also have cardiovascular or metabolic comorbidities, further complicating sedation management [75].

A propofol-sparing, opioid-free anesthesia is recommended to enhance safety. Remimazolam offers minimal respiratory depression and rapid recovery, making it preferable to propofol. Dexmedetomidine provides sedation while maintaining spontaneous ventilation, while (es)ketamine helps preserve airway patency and reduces opioid requirements. Intravenous lidocaine further lowers the need for propofol and opioids, mitigating risks of hypotension and respiratory suppression [75,76]. A study by Li et al. found that remimazolam combined with esketamine resulted in stable hemodynamics, effective sedation, and reduced respiratory depression compared to propofol in outpatient colonoscopy, supporting its use in high-risk populations. It requires further investigation [77].

To prevent hypoxemia and apnea, proactive oxygen supplementation is essential. High-flow nasal oxygen therapy (HFNOT) improves oxygenation and reduces airway collapse, making it particularly suitable for patients with moderate-to-severe obesity and obstructive sleep apnea (OSA) [78]. Unlike HFNOT, nasal CPAP did not prevent hypoxemia in this group of patients and should not be used routinely [79]. Conversely, continuous capnography is strongly recommended in this population for the early detection of apnea, as the risk is inherently increased by both underlying respiratory vulnerability and the depressant effects of most anesthetic agents [80].

Optimizing sedation in this population requires pre-procedure screening for undiagnosed OSA, careful sedative selection, opioid minimization, and advanced oxygenation strategies. Integrating these measures improves patient safety and procedural success, reducing complications associated with sedation-related respiratory depression.

### 6.2. Cardiovascular Diseases

Patients with cardiovascular diseases (CVD) are particularly vulnerable to hypotension during colonoscopy and its associated risks. The selection of an appropriate sedation strategy should account for blood pressure, heart rate, the presence of arrhythmias, and medications being used. In this patient group, it is advisable to employ propofol-sparing strategies, starting with low doses of propofol or combining it with other sedative agents [53,54,55,56].

Remimazolam has shown advantages over propofol in maintaining stable hemodynamics, making it particularly suitable for patients with CVD. Its predictable pharmacokinetics and minimal impact on blood pressure make it an emerging alternative for procedural sedation in high-risk populations [60,81].

Dexmedetomidine, with its sympatholytic properties, helps stabilize heart rate and blood pressure, making it a suitable option for patients with CVD However, it is important to note that dexmedetomidine, when used alone, more frequently causes hypotension and bradycardia compared to propofol [55]. Studies comparing dexmedetomidine-ketamine with propofol-fentanyl for colonoscopy sedation suggest that the first combination results in fewer hemodynamic fluctuations [82].

Ketamine preserves cardiovascular stability by stimulating sympathetic activity, which can counteract the hypotensive effects of other sedatives. When combined with dexmedetomidine, its hypertensive and tachycardic effects are mitigated, offering a balanced sedation profile. Low-dose ketamine, when combined with propofol or dexmedetomidine, can be a safe alternative in this patient group [14,15,55,82].

A propofol-sparing strategy aims to reduce propofol requirements by incorporating adjunct agents such as dexmedetomidine, ketamine, remimazolam, or intravenous lidocaine. These agents not only enhance sedation but also help maintain hemodynamic stability, reducing the risk of hypotension and myocardial depression.

Tailoring sedation protocols to individual cardiovascular risk profiles, with careful dose titration and continuous hemodynamic monitoring, is essential for minimizing complications.

### 6.3. Respiratory Diseases

In patients with respiratory diseases, sedation strategies should prioritize agents with minimal risk of hypoventilation, airway obstruction, and hypoxia. Propofol, dexmedetomidine, ketamine, and remimazolam are preferable, while midazolam and opioids should be avoided or used cautiously. Continuous monitoring of oxygenation, ventilation, and airway patency is essential to ensure patient safety [14,15,17,81,82].

### 6.4. Elderly and Frail Patients

Sedation in elderly and frail patients undergoing colonoscopy necessitates careful consideration due to age-related alterations in pharmacokinetics and pharmacodynamics. Reduced hepatic metabolism and renal clearance contribute to prolonged drug elimination, increasing the risk of accumulation and extended sedation. Furthermore, hypoalbuminemia and cachexia may enhance the effects of protein-bound drugs, leading to heightened drug sensitivity. Increased central nervous system susceptibility further predisposes this population to hypotension, respiratory depression, and delirium, even at standard doses, thereby exacerbating the risk of hemodynamic instability and delayed recovery [14,15,52,53,83].

Several studies have highlighted the need for tailored sedation strategies in elderly patients undergoing colonoscopy. Remimazolam has been shown to offer a safer sedation alternative compared to propofol, with reduced risks of respiratory depression and hemodynamic instability. A meta-analysis by Ahmer et al. found that remimazolam, compared to propofol, provided similar efficacy but with fewer adverse events, particularly in elderly patients, suggesting its role as a preferred agent in this population [34].

Propofol remains a popular choice due to its rapid onset and quick recovery profile. However, its use in the elderly requires careful titration to avoid hypotension and prolonged sedation. In this group, the addition of intravenous lidocaine has been shown to reduce the required dose of propofol, thereby minimizing the risk of hemodynamic instability. A study by Chen et al. demonstrated that lidocaine can act as a propofol-sparing agent, improving sedation while reducing the cardiovascular side effects typically associated with propofol use [57]. Additionally, low-dose ketamine has shown effectiveness in preserving airway reflexes and hemodynamic stability, reducing the need for opioids, propofol and benzodiazepines and their associated risks, including delirium and respiratory depression [15,59,72,82].

Dexmedetomidine is emerging as a promising sedative for elderly and frail patients undergoing colonoscopy. It has shown potential in delirium prevention and offers sedation without significant respiratory depression, making it particularly suitable for this population. However, it is associated with bradycardia and hypotension, complications to which elderly patients, especially those with underlying cardiovascular and respiratory diseases, are more prone. Therefore, dexmedetomidine should be used cautiously, with close monitoring of hemodynamics. In certain cases, it may be combined with low-dose ketamine to achieve hemodynamic stability, especially in patients with preexisting cardiovascular or respiratory conditions [15,34,56,84].

## 7. Patient Monitoring During Colonoscopy

### 7.1. Basic Physiological Monitoring

Vigilant patient monitoring is a cornerstone of safe procedural sedation during colonoscopy, in alignment with recommendations from leading international bodies [52,85]. Standard monitoring is essential for the early detection and mitigation of cardiorespiratory adverse events. This typically includes continuous evaluation of patient oxygenation via pulse oximetry, hemodynamic stability through non-invasive blood pressure (NIBP) measurements, and cardiac rhythm assessment with electrocardiography (ECG) [86]. Furthermore, professional guidelines, including those from the American Society of Anesthesiologists (ASA), increasingly recommend the use of capnography for monitoring ventilation [85,87]. The addition of end-tidal carbon dioxide (EtCO2) monitoring provides an earlier warning of respiratory depression and apnea compared to pulse oximetry alone, and its implementation has been shown to significantly reduce the incidence of hypoxia and other adverse events during sedation [87,88,89]. The routine administration of supplemental oxygen is also a common practice to enhance the margin of safety by preventing or lessening the severity of hypoxemia should it occur.

### 7.2. Depth of Sedation Monitoring

The precise assessment of sedation depth during colonoscopy has evolved significantly with the introduction of processed electroencephalographic monitoring systems. The Bispectral Index (BIS) represents the most extensively validated neurophysiological parameter, processing raw EEG data to generate values between 0 and 100, where 60–80 indicates appropriate sedation levels for endoscopic procedures [90]. The Patient State Index (PSI), generated by the Masimo SedLine^®^ system, provides a comparable measure of sedation depth, where values of approximately 75 indicate moderate sedation [91], 50–70 signify deep sedation [92], <50 suggests general anesthesia, and <25 indicates a burst suppression pattern [93]. While many procedures can be performed under moderate sedation, colonoscopy often requires deep sedation to ensure patient comfort and procedural success [94]. Subjective sedation scales correlate well with processed EEG indices like BIS and PSI, supporting their complementary use for sedation monitoring during colonoscopy. For example, BIS values showed a strong correlation with Ramsay scores (rho = −0.73; *p* < 0.001), validating EEG monitoring as an objective adjunct to clinical sedation assessments [67].

In addition to processed EEG, clinicians widely use subjective sedation scales to titrate medication and assess patients. Commonly used instruments include the Ramsay Sedation Scale [95], the Richmond Agitation-Sedation Scale (RASS) [96], and the Modified Observer’s Assessment of Alertness/Sedation (MOAA/S) scale [97].

A landmark prospective observational study of 119 outpatients undergoing colonoscopy at Thomas Jefferson University Hospital utilized PSI monitoring to assess sedation depth during propofol administration [93]. This investigation revealed alarming findings regarding the actual depth of sedation achieved during what providers intended as moderate sedation.

Clinical investigations have demonstrated that BIS-guided sedation significantly improves cognitive preservation compared to conventional assessment methods. A randomized controlled trial involving 100 colonoscopy patients showed superior postprocedural cognitive function in the BIS-monitored group, with higher Mini-Mental State Examination scores and Clock Drawing Test performance [90]. However, the clinical utility varies across contexts, with some studies paradoxically showing increased propofol consumption in BIS-guided groups compared to clinical assessment alone [98]. This seeming paradox may be explained by the primary goal of BIS monitoring: maintaining a stable and consistent depth of sedation. By providing an objective measure of brain activity, BIS allows clinicians to titrate drug administration precisely to avoid deep sedation troughs, which are a key risk factor for cognitive impairment [99]. Therefore, while the total dose of propofol may sometimes be higher to prevent fluctuations into lighter sedation levels, the ultimate benefit is enhanced patient safety and better preservation of cognitive function.

The clinical benefits of neurophysiological monitoring extend beyond drug optimization to encompass improved patient outcomes. Multiple investigations have consistently demonstrated preserved postprocedural cognitive function, enhanced hemodynamic stability, and reduced incidence of complications. Studies comparing different sedation regimens have shown that propofol-based combinations guided by BIS monitoring provide superior cognitive preservation compared to traditional benzodiazepine-opioid combinations [90,100].

## 8. Conclusions

Optimizing sedation for colonoscopy requires a patient-centered approach that balances efficacy, safety, and recovery. By integrating evidence-based practices and leveraging emerging pharmacological agents, clinicians can enhance patient comfort, improve procedural outcomes, and reduce the burden of anesthesia-related complications. Future research should focus on long-term outcomes of novel sedation strategies and their impact on patient adherence to colorectal cancer screening and surveillance programs.

## Figures and Tables

**Table 1:** Factors to Consider When Qualifying a Patient for Colonoscopy Under Deep Sedation.

Patient-Related Factors	Other Factors
Chronic opioid use Susceptibility to nausea and vomiting Previous unsatisfactory experience with moderate sedation (e.g., inadequate pain control or recall of the procedure) History of sexual abuse Comorbidities (e.g., irritable bowel syndrome, fibromyalgia, colonic diverticulosis, inflammatory bowel diseases) Children and adolescents Cognitive disorders (e.g., dementia, intellectual disability) Strong personal preference for deep sedation	Expected difficulty during examination Planned longer or more complex endoscopic procedures (e.g., therapeutic interventions, polypectomy, or stricture dilation) Limited experience of the physician performing the examination Availability of anesthesia providers and monitoring/emergency equipment

**Table 2:** Propofol dosing in continuous infusion for colonoscopy.

Indication	Initial Dose	Maintenance Infusion	Effect-Site Concentration (Ce)
General Population	1–2.5 mg/kg IV	25–100 µg/kg/min IV	
Elderly or Frail Patients	0.5–1 mg/kg IV	10–50 µg/kg/min IV	2–2.5 µg/mL *
Adjunct with Opioids	0.5–1.5 mg/kg	10–75 µg/kg/min IV	

* Target-controlled infusion (TCI).

## Abbreviations

The following abbreviations are used in this manuscript:

ASA	American Society of Anesthesiologists
BIS	Bispectral Index
BMI	Body Mass Index
CO_2_	Carbon Dioxide
CGRP	Calcitonin Gene-Related Peptide
COPD	Chronic Obstructive Pulmonary Disease
EMR	Endoscopic Mucosal Resection
ESD	Endoscopic Submucosal Dissection
GI	Gastrointestinal
MAC	Minimum alveolar concentration
MOAA/S	Modified Observer’s Assessment of Alertness/Sedation
NMDA	N-Methyl-D-Aspartate
OIH	Opioid-Induced Hyperalgesia
OSA	Obstructive Sleep Apnea
PSI	Patient State Index
TCI	Target-Controlled Infusion
